# Exploring stem cell frontiers: definitions, challenges, and perspectives for regenerative medicine

**DOI:** 10.1242/bio.060245

**Published:** 2024-04-09

**Authors:** Miriana Dardano, Tamina Lebek, Ingrid H. C. Tsang

**Affiliations:** ^1^ Leibniz Research Laboratories for Biotechnology and Artificial Organs (LEBAO), Department of Cardiothoracic, Transplantation and Vascular Surgery, Hannover Medical School, Hannover 30625, Germany; ^2^ Centre for Regenerative Medicine, Institute for Regeneration and Repair, School of Biological Sciences, The University of Edinburgh, Edinburgh EH16 4UU, UK; ^3^ Novo Nordisk Foundation Center for Stem Cell Medicine (reNEW), University of Copenhagen, Copenhagen N DK-2200, Denmark

**Keywords:** Pluripotency, Regenerative medicine, Stem cell therapies, Stem cells, Summer school

## Abstract

Each year, the European Summer School on Stem Cell Biology and Regenerative Medicine (SCSS) attracts early-career researchers and actively practicing clinicians who specialise in stem cell and regenerative biology. The 16th edition of this influential course took place from 12th to 19th September 2023 on the charming Greek island of Spetses. Focusing on important concepts and recent advances in stem cells, the distinguished faculty included experts spanning the spectrum from fundamental research to clinical trials to market-approved therapies. Alongside an academically intensive programme that bridges the various contexts of stem cell research, delegates were encouraged to critically address relevant questions in stem cell biology and medicine, including broader societal implications. Here, we present a comprehensive overview and key highlights from the SCSS 2023.

## Teaser

**Summary:** This Meeting Review explores cutting-edge research and its translational potential discussed at the 16th European Summer School on Stem Cell Biology and Regenerative Medicine (Spetses, Greece, 12th–19th September 2023).

## Introduction

**Fig. 1.**
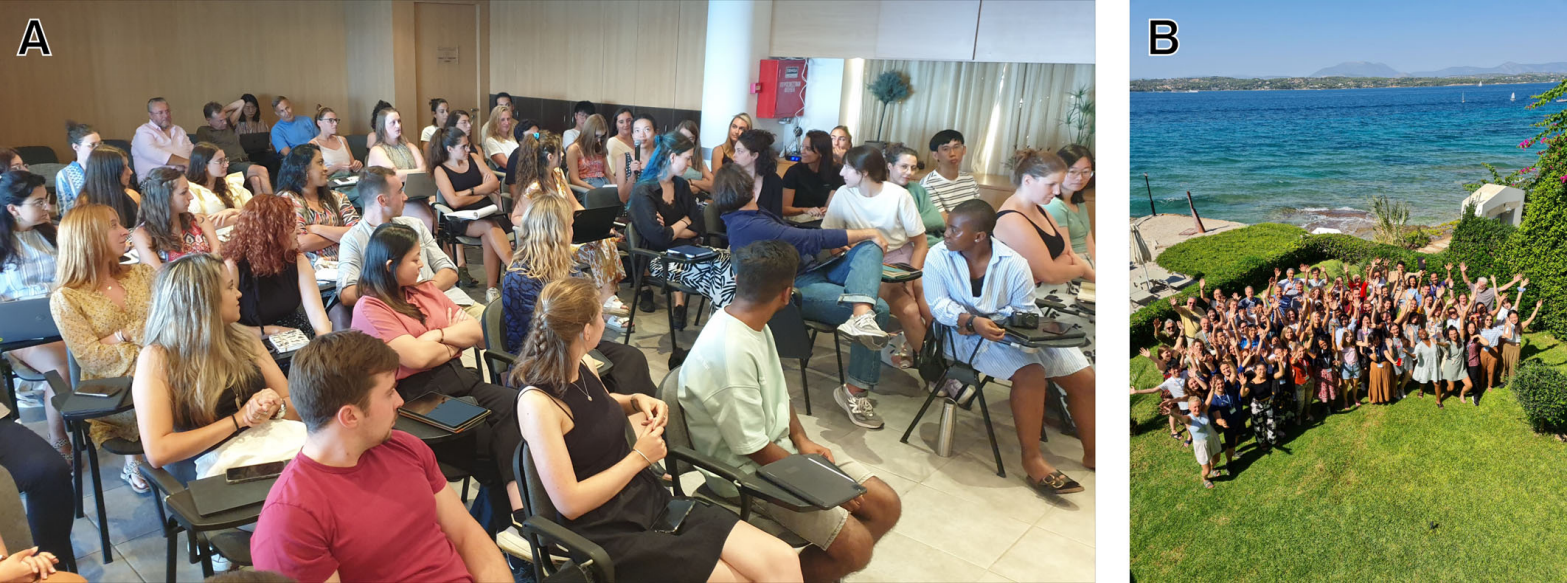
**People of the SCSS 2023.** (A) Attendees discussing the importance of defining ‘stem cells’ for researchers and the public. Photo credit Sally Lowell. (B) The attendees and faculty of the SCSS 2023 at the beach of the Spetses Hotel. Photo credit Jenny Nelder.

The European Summer School on Stem Cell Biology and Regenerative Medicine (SCSS; formerly Hydra Summer School, https://www.stemcellsummerschool.org/) was initially founded in 2005 to facilitate the training of young scientists’ abilities to think critically about fundamental questions underlying stem cell research, developmental biology, and regenerative medicine. Importantly, delegates were tasked with articulating broad topics such as ‘what is a stem cell?’ – a particularly salient question against the backdrop of controversies in the 2000s within the stem cell field around different kinds of stem cells, and whether a ‘universal’ one existed. Since then, the field also had to grapple with additional questions around ‘what is or is *not* a stem cell therapy?’. SCSS has continued to tackle current questions in stem cell research, and the summer school has grown to be a key training ground for mature PhDs, postdocs, clinicians, and other early career researchers (ECRs) around the world (Fig. 1). Now, in addition to definitions of this field's foundational concepts, SCSS attendees were asked to consider why and how debating fundamental questions in stem cell biology is essential for the sustainability of regenerative medicine.

In this Meeting Review, we present the discussions and perspectives the 2023 summer school offered regarding current, state-of-the-art stem cell research, with an emphasis on those especially relevant to the ECR community. For the first time in its 18-year history, SCSS 2023 was held on the Greek Island of Spetses instead of the neighbouring island of Hydra (after which it was formerly named). We begin by providing an overview of the interactive sessions that guided participants in thinking critically about the field. We then synthesise key themes that emerged from faculty-led lectures, many of which included unpublished research. Finally, we reflect on the significance of this meeting for us as SCSS 2023 delegates, and the potential impact we believe it has for other ECRs in the stem cell field.

## Interactive sessions

**Fig. 2.**
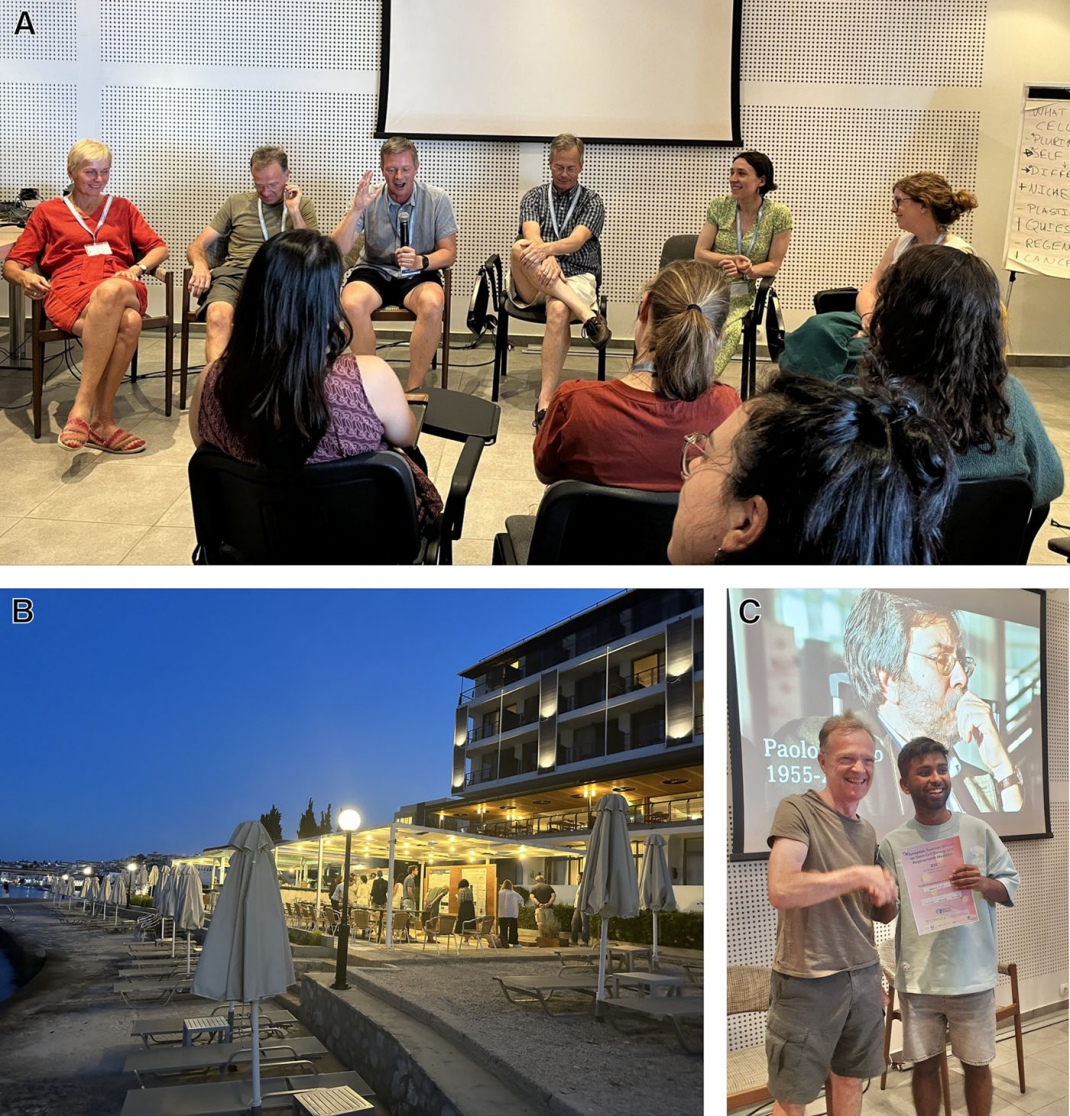
**Interactive sessions at the SCSS 2023.** (A) Career session with (left to right) Christine Mummery, Austin Smith, Kim Jensen, Jim Wells, Elisa Laurenti, and Sally Lowell. Photo credit Giuseppe Calà. (B) Poster session at the beach of Spetses Hotel. Photo credit Kim Jensen. (C) Austin Smith with Paolo Bianco Prize winner Indranil Singh. Photo credit Sally Lowell.

A defining feature of SCSS is the amount of care taken to maximise opportunities for both reflections on current research progress and reflexivity around being a stem cell scientist. Interactive sessions are a vital part of this effort by bringing the SCSS's diversity of participants into productive dialogue with each other. Comprising a wide range of activities, interactive sessions made up almost 50% of the 2023 summer school's programme (Fig. 2).

In accordance with the summer school's traditions, SCSS 2023 opened with a debate session: ‘What is a stem cell?’ with the aim of converging on a consensus. While we quickly reached a collective agreement that there is no ‘universal’ stem cell and it is important to talk about *kinds* of stem cells in different systems and contexts, we did not manage to articulate a singular common thread that exhaustively and exclusively captured them within the allocated time. This sentiment of diversity echoed throughout the lectures of the summer school, and so the closing debate revisited this topic with an additional question: ‘Does having a consensus definition around the term ‘stem cells’ matter?’. Dominant points of discussion surrounded the need for consensus in science communication and education.

The themes of scientist's roles in society and scientific research culture were also prominent in other debate sessions, where delegates democratically decided to tackle questions from ‘What are the different scales of health inequalities and what can researchers do to address them?’ to ‘How does the balance between competition and co-operation affect science and where should this balance lie?’. These sessions were important in encouraging the participating ECRs to reflect on bigger pictures of how science should be conducted, and what impacts it has in the wider world.

In addition to the far-reaching impacts of science, the summer school also invited delegates to delve into the finer details of current research efforts. Each lecture was followed by a small group discussion session with the presenting faculty, which were delegate-led opportunities to hear behind-the-scenes insights from their research. ‘Revisiting the lectures’ sessions were also held, whereby key take-home messages of each lecture and the new questions they raised were identified by small groups of participants and fed back to the others. These provided a further stimulating chance for ECRs to get to know each other's thought-processes and encouraged the formation of collaborations.

Networking and creating connections are among the most important aspects of building a successful scientific career. Organisers seemed acutely aware of this, as there was a strong focus on facilitating constructive interactions between all attendees. Faculty members were often present in each other's lectures and participated in question-and-answer sessions on equal terms with ECRs, fostering a true sense of openness and community. The programme also included a careers session, where a panel of speakers shared highlights from their personal lives and careers, offering good advice on how to advance a scientific career.

The SCSS also took full advantage of its beautiful surroundings in the Greek Saronic Islands to break the formal hierarchy that often separates junior and senior researchers in conference halls and lecture theatres. Each delegate presented their research in poster sessions spread out across four evenings on a private beach. Here, in between enjoying finger foods in the summer evening breeze, we openly exchanged suggestions and observations with each other, and received feedback from faculty about the succinctness of our poster presentations (which were intended to be limited to two minutes). Another highlight was a networking boat trip to the picturesque beach of Zogeira, where all summer school participants connected over a seaside lunch and made the most of the Mediterranean weather.

## Lectures

### Fundamental stem cell research: past, present and future

Integral to the SCSS are lectures that delve into the past, present, and future of fundamental research, all around the dynamics of stem cells. Notably, in two key lectures, Austin Smith (Living Systems Institute at the University of Exeter, UK) gave both an overview of the history of stem cell research and an insight into debates on the use of mouse versus human pluripotent stem cells (PSCs) in modelling development, emphasising the nuanced similarities and differences in timing and potency (Guo et al., 2021; Kinoshita et al., 2021; Smith, 2017). These lectures introduced the stem cell concept focusing on the pluripotency criterium, the potential of a single cell to generate multiple fates by reacting to stimuli. This was demonstrated at the dawn of stem cell research by single cell transplantation of embryonal carcinomas (Kleinsmith and Pierce, 1964). The protocols developed using these cancer stem cells laid the groundwork for culturing embryonic stem cells with the capacity for long-term self-renewal and for contributing to all three germ layers in intraspecies chimeras – the two defining features for mouse embryonic stem cells. While the work on human embryonic stem cells is entirely based on the concepts of mouse embryonic stem cells, we cannot test for pluripotency by creating human chimeras for ethical and technical reasons, challenging researchers to develop new strategies and concepts for stem cell research.

New strategies to evaluate the lineage potential of stem cells are driven by recent advances in single cell analysis. Allon Klein (Harvard Medical School, MA, USA) contributed to the discourse about the stem cell concept with insights into cell types and states based on LARRY, a novel DNA-barcoding strategy for analysing clonal cell fates using single-cell RNA sequencing (Weinreb et al., 2020). Challenging our conventional understanding, his talk culminated in the provocative idea that stem cells represent a behaviour rather than a distinct cell type with cellular plasticity being key for stem cell behaviour.

Sally Lowell (Centre for Regenerative Medicine, Institute for Regeneration and Repair, University of Edinburgh, UK) underscored the significance of mapping cell behaviour in relation to neighbouring cells to comprehend development and disease, highlighting how recent advancements in neighbour-labelling systems have revolutionised this rapidly growing research field of listening in on cellular crosstalk (Lebek et al., 2023 preprint; Malaguti et al., 2022).

Several lectures then spotlighted the importance of basic research to understand regeneration across different organ contexts. Elisa Laurenti (Wellcome–MRC Cambridge Stem Cell Institute at the University of Cambridge, UK) ignited a discussion on blood as a regenerative powerhouse, and how the dynamics of hematopoietic stem cells change with age (Mitchell et al., 2022). Joo-Hyeon Lee (Wellcome–MRC Cambridge Stem Cell Institute at the University of Cambridge, UK) detailed her group's exploration of lung plasticity during alveolar regeneration, where lung cells undergo trans-differentiation after injury to restore tissue homeostasis (Choi et al., 2020, 2021). Meanwhile, Shahragim Tajbakhsh (Pasteur Institute, France) provided insights into the complexity of muscle stem cells in different parts of the body and at different stages in life, highlighting the transcriptional and epigenetic consequences of cellular origin and age on the stem cell potential (Esteves de Lima et al., 2021; Hernando-Herraez et al., 2019). Cedric Blanpain (Université libre de Bruxelles, Belgium) gave two illuminating talks on cell competition and plasticity in skin (Dekoninck and Blanpain, 2019; Pérez-González et al., 2023), and the intriguing parallels between mammary gland and prostate revealed through lineage tracing experiments (Centonze et al., 2020).

Last but not least, Elly Tanaka (IMP Research Institute of Molecular Pathology, Austria) rounded off the lectures on fundamental stem cell research by introducing the remarkable axolotl as an invaluable model organism for studying development and regeneration (Gerber et al., 2018; Lust et al., 2022; Schloissnig et al., 2021), emphasizing the importance of exploring biological extremes in nature to unravel essential processes such as regeneration – maybe cellular mechanisms and tissue behaviours discovered in the salamander model can be translated into biomedical strategies applicable in the field of regenerative medicine.

### Advances towards the clinic: drug screening, disease modelling and therapeutic proof-of-concepts

Beyond the stimulating talks on stem cell biology fundamentals, we also attended enlightening lectures from experts leading research aiming to create *in vitro* systems for drug-screening and disease modelling, paving the way for novel therapeutic approaches. Kim Jensen (NNF Centre for Stem Cell Medicine, University of Copenhagen, Denmark) gave an interesting lecture about the intestine's physiology, crypts composition, and the *LGR5+* intestinal epithelial stem cells (Guiu et al., 2019). Such stem cells have the competence to grow organoids *in vitro*, which can be used for transplantation and as models for addressing key physiology-related biological questions (Watanabe et al., 2022). James Wells (Cincinnati Children's Hospital Medical Centre, OH, USA) presented his research on developing highly intricate PSC-derived organoids for studying intestinal development and pre-clinical testing (McCracken et al., 2014; Spence et al., 2011). By transplanting and culturing these human intestinal organoids in humanised mice, the Wells Lab achieves unprecedented size and complexity, pioneering a new avenue for investigating the crucial interplay between the gut and the immune system (Bouffi et al., 2023; Watson et al., 2014). Concluding the theme of intestinal biology, Matthias Lutolf (Roche Institute of Human Biology, Basel, Switzerland) presented the progress of his group in bioengineering intestinal ‘multi-tissue systems’. In contrast to the self-patterning organoids used in the Jensen and Wells Labs, his group generates their organoid systems by seeding stem cells onto hydrogel-based scaffolds that mimic the intestine's epithelial shape, allowing the derivation of self-organised mini-intestines on a chip. He then showed that it is possible to increase the complexity of this system by inducing tissue vascularization or by adding immune cells to the scaffold (Mitrofanova et al., 2023 preprint; Nikolaev et al., 2020).

Another very important organ to model *in vitro* is the heart and Christine Mummery (Leiden University Medical Centre, Netherlands) is a leading expert in this field (Arslan et al., 2022). In the Mummery Group, cardiac micro-tissues are generated by either differentiating and mingling different heart-forming cell types or differentiating all the cell types of interest simultaneously with an appropriate 3D-culture protocol (Arslan et al., 2023). The scientific progresses addressed by these four speakers are incredibly important to understand the physiology of the respective *in vitro* modelled organs, providing potent drug-screening platforms and *in vitro*-derived tissues for cell replacement therapies.

A somewhat controversial but extremely interesting talk was given by Hiromitsu Nakauchi (Stanford University School of Medicine, CA, USA, and Institute of Medical Science, University of Tokyo, Japan) whose group focusses on growing organs of one species in another, creating interspecies chimeras (Kobayashi et al., 2010; Nishimura et al., 2021). The principle already works between closely related species such as rat and mouse. The therapeutic potential is clear; the technology envisages generating hiPSCs from an organ transplant candidate and integrating these cells into a blastocyst of an animal, ideally a pig, with disabled development of a particular organ, e.g., restricted pancreatogenesis for growing pancreas from hiPSCs. The patient-derived organ will then grow together with the host animal and, upon maturation, could potentially be extracted for transplantation (Kano et al., 2022). Growing human organs in host animals opens new perspectives in the field of regenerative medicine, enabling the growth of organs on-demand and addressing the challenges of organ shortages and transplant rejections. However, limitations due to interspecies xeno-barriers still need to be addressed, as do the ethical concerns arising from such techniques (which were also discussed during the debate sessions).

As an expert in the cancer biology field, we were delighted to virtually host Steven Pollard, (Centre for Regenerative Medicine, Institute for Regeneration and Repair, University of Edinburgh, UK) who presented an enlightening methodology to selectively target solid tumour cells, using the highly aggressive glioblastoma (GBM) as a proof-of-concept model (Castillo et al., 2023; Gangoso et al., 2021). The technology consists of ingeniously utilising the grammar-like way in which specific transcript factors (TFs) bind enhancers of specific genes, enabling a selective and controlled expression of proteins of interest (such as payloads and cytotoxins) in specific cell types that express the appropriate combination of TFs (such as the GBM cancer cells). The technique is already curative in an aggressive mouse glioblastoma model and opens incredible clinical possibilities for targeting GBM and, potentially, other solid tumours.

### Into the clinic and beyond: trials and tribulations of translating stem cell research into regenerative medicine

In addition to the talks on pre-clinical research, we also had the privilege to hear about stem cell therapies that are either in ongoing clinical trials, or that have already been market approved. A key theme across them was the synergy between basic stem cell research and translational endeavours. Instead of presenting this relationship as a one-way street from the former to the latter, all speakers took great efforts to emphasise that continuous feedback between the two is necessary.

In the opening keynote, Michele De Luca (Stefano Ferrari Centre for Regenerative Medicine, University of Modena and Reggio Emilia, Italy) chronicled the development and progress of Hologene 5 – a cell therapy that is currently in clinical trials for junctional Epidermolysis Bullosa (JEB) (De Rosa et al., 2021). In addition to the success achieved in these clinical trials (Bauer et al., 2017; Hirsch et al., 2017; Mavilio et al., 2006), he also reflected on different instances of ‘failure’ and how they each provided insight into fundamental biological processes, such as the competition dynamics of epidermal stem cells. In a subsequent session, Michele De Luca presented Holoclar – a limbus stem cell treatment for ocular burns and one of the few advanced therapy medicinal products (ATMP) approved by the European Medicines Agency (EMA). He then discussed various ATMP-specific obstacles both before and after EMA-approval. His talk concluded with a very emotional reminder of what these therapies mean to patients, and the devastation caused by withdrawing these therapies on financial grounds.

Agnete Kirkeby (NNF Centre for Stem Cell Medicine, University of Copenhagen, Denmark) then shared some very exciting progress in her work developing regenerative therapies for neurodegenerative diseases. She primed us with an initial lecture demonstrating how results in neurodevelopmental biology can inform the finessing of neuronal cultures (Kirkeby et al., 2012; Rifes et al., 2020). She weighed in on the debate over whether 2D or 3D *in vitro* models were better (2D for reproducibility and scalability) and emphasised that she believes ‘there is no such thing as a universal neural stem cell’ (despite what textbooks may say). This was followed by a second comprehensive presentation covering both the current state of the STEM-PD trial in Europe (Kirkeby et al., 2017, 2023) and hPSC-based clinical trials more globally.

Finally, in the closing keynote Pete Coffey (Institute of Ophthalmology, University College London, UK) updated us on the London Project to Cure Blindness (Coffey, 2019; da Cruz et al., 2018). He started by giving an impressively succinct introduction to the basic biology underpinning both retinal cell dynamics and retinopathies. He then discussed the project's endeavours to treat age-related macular dystrophy (AMD) using a transplantable retinal pigment epithelium (RPE) graft, and described occasions where operating on human patients necessitated the research team to innovate in unexpected ways (Coffey et al., 2020).

A unique aspect of this summer school was how comfortable and open the faculty were with sharing their insights and experiences. All three faculty members who presented clinical trials impressed on us the practical considerations necessary to translate their work into approved therapies. Agnete Kirkeby went through the paperwork and bureaucracy required in this process, Pete Coffey made a point of showing how the patient's day-to-day lives can unexpectedly affect clinical outcomes during the trials, Michele De Luca stressed the financial requirement for approved therapies to stay in the market. Overall, we were fortunate enough to receive a concise overview of what it takes to bring candidate therapies through clinical trials, into the market and keep them there.

## Cultures and infrastructures of stem cell research

As stem cell research advances towards the clinic, it is imperative that we also consider the infrastructures necessary for reproducible and/or standardised outputs. Presentations from Glyn Stacey and Andreas Kurtz (respectively Director and Co-director of The International Stem Cell Banking Initiative, UK) underscored the critical role of ensuring the health, stability, and authenticity of cell lines in the interpretation of cell culture data. Their insights shed light on the regulatory aspects and challenges associated with stem cell banking, particularly in the context of human pluripotent stem cells (Mah et al., 2020; Stacey and Healy, 2021).

Amidst the technically intensive scientific discussions, Jan Barfoot (University of Edinburgh, UK) and Furkan Karayal (founder of Diverse In) provided refreshing talks about the importance of different kinds of communication for science research. Their presentations highlighted the significance of inter-personal communication and awareness to foster an accessible, diverse, and inclusive environment in scientific discourse. They also emphasised the value of public engagement, not only for personal growth but also for the advancement of the scientific community and society at large.

## Discussion

In this summer school, we had the opportunity to enrich our knowledge in the field of stem cell biology. Stem cells are the basis of complexly organised life forms, from the first PSCs able to generate a whole organism to the tissue-specific stem cells enabling tissue regeneration in the adult. Largely underpinned by the faculty members was that several different types of stem cells exist and can be distinguished at different levels: on the base of their potency, metabolic activity, anatomical location, renewal capacity and more. Considering the heterogeneity of stem cells, it is challenging to find a common definition accommodating every single category. Yet, we left the summer school with the conclusion that fundamental threads can be identified to harmonise investigations into the nature of these wondrous cells.

**Fig. 3.**
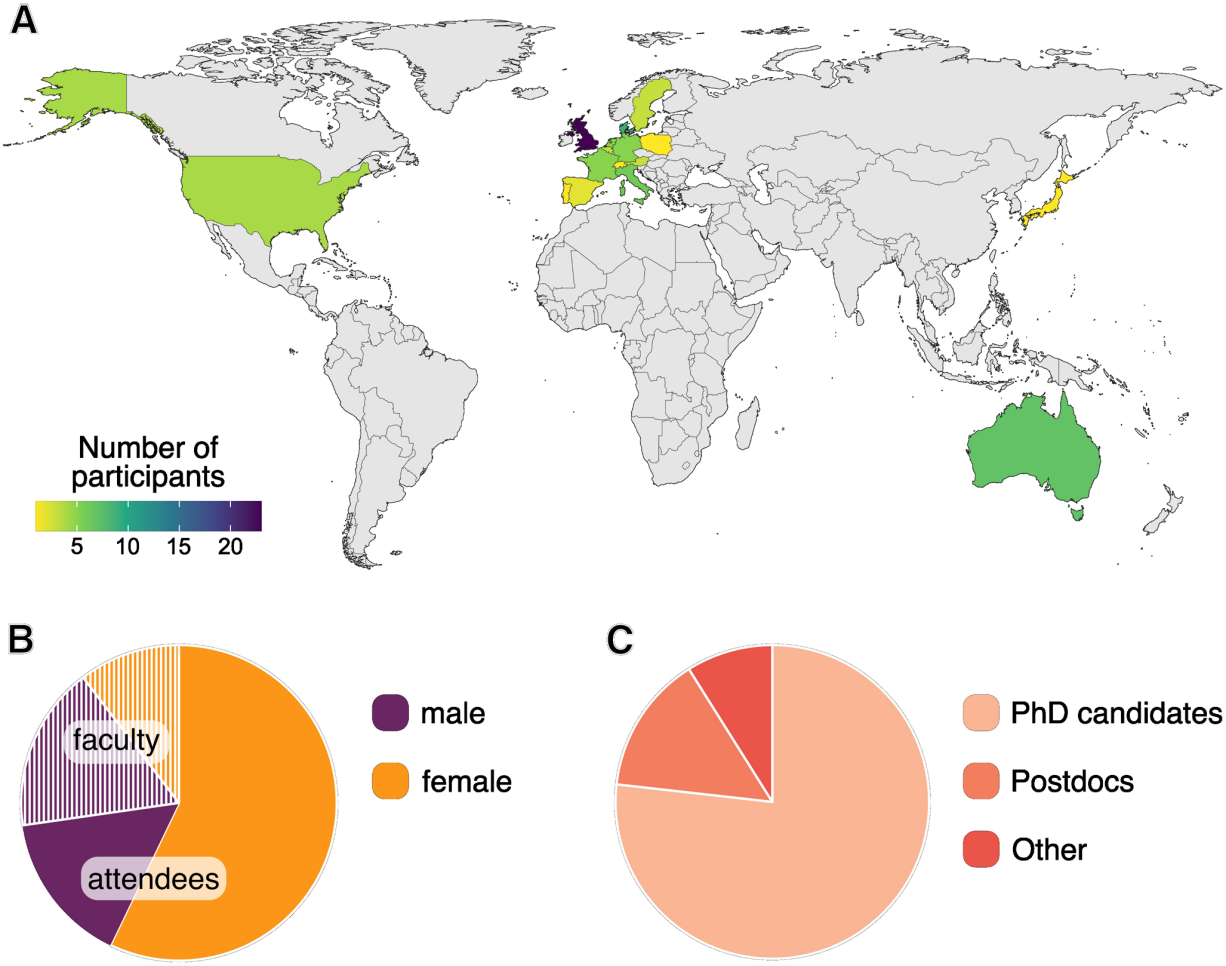
**Demographics of the SCSS 2023.** (A) The SCSS 2023 welcomed attendees and faculty from around the globe. (B) There was an overwhelming majority of female attendees at this SCSS, with a very balanced gender ratio among the faculty members. (C) The SCSS is aimed at early career researchers in the field of stem cell biology with most of the attendees being PhD candidates in 2023.

Astounding progress has been made within the stem cell field in the last two decades. Over the course of just one week, we have also been able to meditate on the academic progress of stem cell biology, the societal impact of regenerative medicines, and our personal roles in this (Fig. 3). We had the pleasure of attending talks by the experts that led such advancements and are now translating their knowledge to treat currently incurable diseases. This cutting-edge research was not the only highlight of this summer school. Building on these lectures, the summer school's plentiful interactive sessions also offered invaluable opportunities to create connections with faculty members and other attendees (indeed, the writing of this Meeting Report is evidence of this great network). Finally, we feel that the SCSS 2023 was a pivotal experience because it guided us in collectively reflecting on key topics such as how stem cell research should be conducted in accordance with societal, academic, ethical, and personal responsibilities. A central discussion theme was the communication of stem cell-related knowledge from the bench to the public, since the ultimate aim of our research is to develop stem cell-based therapeutic strategies for the public itself. To conclude, a thought-provoking quote from Austin Smith captures such a challenging yet crucial theme:

‘We can always justify our research to ourselves, but can you justify it to your mum and dad? Your partner?’
